# Transactions Medical Association of Georgia

**Published:** 1882-02-20

**Authors:** 


### *TRANSACTIONS MEDICAL ASSOCIATION OF GEORGIA.*

Thirty-second annual session, at Thomasville, Ga., April, 1881. Edited by A. Sibley Campbell, M. D., Secretary Augusta, Ga.

We are indebted to Dr. Campbell, the secretary, for a copy of the above, a book of 314 large octavo pages, gotten up in large plain type, neatly arranged illustrated and well executed in every department, evincing great skill, taste, energy and ability on the part of Dr. Campbell.

In addition to the business proceedings of the soeiety, it contains the by-laws and constitution; a roll of its living and deceased members, President LeHardy's excellent address and a number of able and interesting papers. We regret the want of space to make special mention as to the merits of the several articles. The following are the names of the contributors to the volume, to-wit:

J. C. LEHARDY, M. D., President

R. J. Nunn, M. D.; Thomas R. Wright, M. D.; Thomas H. Kenan, M. D.; J. G. Hopkins, M. D.; W. H. Philpot, M. D.; A. Sibley Campbell, M. D.; Charles W. Hickman, M. D.; S. B. Hawkins, M. D.; DeSaussure Ford, M. D.; S. H. Stout, M. D.; W. F. Westmoreland, M. D.; K. P. Moore, M. D.; T. S. Hopkins, M. D.; Henry F. Campbell, M. D.; T. O. Powell, M. D.; J. M. Toner, M. D.; L. B. Alexander, M. D.; H. H. Carleton, M. D.; P. H. Wright, M.D.; A. A. Smith, M.D.; A. G. Whitehead, M.D.

The following are the officers of the Association:

Wm. F. Holt, M. D., of Macon, President.<br>

Eugene Foster, of Augusta, }<br>

T. M. McIntosh, of Thomasville, } Vice-Presidents.<br>

A. Sibley Campbell, Augusta, Secretary.<br>

K. P. Moore, Forsyth, Treasurer.<br>

J. R. Duggan, Macon, Orator.

*Board of Censors*—C. A. Hall, Macon; G. W. Holmes, Rome; W. B. Wells, Red Clay; E. L. Connally, Atlanta; R. J. Nunn, Savannah.

